# Unmet health-related social needs are associated with pain outcomes among midlife and older adults

**DOI:** 10.3389/fpain.2026.1885908

**Published:** 2026-09-01

**Authors:** Victoria H. Davis, Elizabeth J. Brines, Mary R. Janevic

**Affiliations:** Department of Health Behavior and Health Equity, University of Michigan School of Public Health, Ann Arbor, MI, United States

**Keywords:** chronic pain, food access, housing, middle-aged adults, older adults, social determinants of health, social needs, social support

## Abstract

**Introduction:**

Chronic pain affects over one-third of U.S. older adults, and particularly socioeconomically constrained populations. Few studies examine how unmet social needs related to the social determinants of health, including food, housing, utilities, transportation, and social support, are linked to pain. This study assessed whether cumulative and individual unmet social needs are associated with pain outcomes in a predominantly Black/African American sample of adults aged 50 years and older with chronic pain recruited from an underserved urban setting.

**Methods:**

We pooled pre-intervention data from two randomized controlled trials of community health worker-led behavioral interventions. Multivariable linear regression examined associations of cumulative and individual unmet social needs with pain intensity, pain interference, and pain self-efficacy.

**Results:**

Among 438 participants, the mean age was 67.5 years, 87% (*n* = 380) were female, 77% (*n* = 338) identified as Black/African American, and 54% (*n* = 237) reported one or more unmet social needs. In adjusted regression models, participants with greater cumulative unmet social needs (three or more) experienced greater pain intensity (*B* = 0.75, *p* = .010), pain interference (*B* = 3.24, *p* = .002), and lower pain self-efficacy (*B* = −2.04, *p* = .006). Food access challenges were associated with all three outcomes; transportation difficulties were linked with greater pain interference and lower self-efficacy; and insufficient social support was associated with greater pain intensity and greater pain interference (*p* < .05). Housing and utility payment difficulties were not associated with pain outcomes.

**Conclusion:**

Cumulative and individual unmet social needs are associated with worse pain outcomes among midlife and older adults with chronic pain.

## 1 Introduction

Chronic pain prevalence among U.S. adults 65 years and older has risen from 30.5% in 2021 to 36.0% in 2023, a larger increase than in any other age group (1). In this population, chronic pain [pain lasting for over 3 months (2)] is associated with impaired mobility and physical functioning (3), anxiety and depression (4), cognitive impairment (5), frailty (6), loneliness (7), and mortality (8, 9). Despite this substantial and increasing hardship, chronic pain remains widely underassessed and undertreated (10, 11), with the heaviest burden often falling on those most affected by structural inequities, including racism and poverty (12–15). These avoidable differences in pain prevalence, management, and treatment are dynamic and stem from multiple intersecting historical and present-day inequalities in power, access to opportunities and resources, wealth, and healthcare (14).

A growing body of work has called for attention to the intersecting and dynamic social factors that shape pain prevalence, experience, and management over the life course (16, 17). Research on the social predictors of chronic pain has largely focused on distal indicators, including education, socioeconomic status, and neighborhood environments. For example, a socioeconomic gradient has been identified whereby lower education predicts more frequent pain and greater pain severity and interference (18). Other studies have reported that living in areas with fewer socioeconomic opportunities is associated with higher mean pain interference (19, 20) and greater frequency of chronic pain among adults (20, 21). Persistent exposure to socioeconomic inequity across the life course, such as working in manual labor, further elevates the odds of pain in older age (22).

Comparatively less is known about more proximal, individual-level social needs, which are the daily non-medical requirements for living [e.g., food, housing, utilities, transportation, and social support (23)] that arise from upstream social determinants of health. These needs represent one way in which broader structural inequities are translated into individual-level health consequences such as pain (17). For example, a nationally representative cross-sectional study found that food insecurity was associated with higher odds of chronic pain after accounting for socioeconomic factors (24). Research has also demonstrated a link between social isolation and increased pain interference with activities of daily living (25). One potential pathway to explain these relationships is chronic stress: prolonged exposure to psychosocial stressors, such as those caused by unmet social needs, can increase vulnerability to chronic pain (26, 27), consistent with the weathering hypothesis (28). However, to our knowledge, studies have not examined how a wider array of cumulative and individual unmet social needs relate to pain outcomes. Identifying these associations can inform modifiable, non-medical targets for more equitable pain interventions and help providers and policymakers prioritize non-medical contributors to pain in this population.

The present study examines the associations between unmet health-related social needs and multiple pain outcomes among midlife and older adults with chronic pain living in an underserved urban community. We assessed the prevalence of unmet social needs and evaluated whether the cumulative number of unmet social needs and individual challenges with food, housing, utilities, transportation, or social support are associated with pain outcomes.

## 2 Method

### 2.1 Study sample

We pooled pre-intervention data from two randomized controlled trials of community health worker-led behavioral interventions for adults aged 50 years and older living in or near Detroit, Michigan. The first (Seniors using Technology to Engage in Pain Self-management, or STEPS) focused on chronic pain self-management (*n* = 193) (29). The second (Re-engaging in Self-care, Enjoying Today, or RESET), from which we drew a subset of participants reporting chronic pain at screening (*n* = 245), tested a positive psychology intervention to enhance psychosocial functioning. Participants were included in the present analysis for STEPS and the RESET subsample if they completed the social needs screener, reported experiencing pain in their muscles or joints on most days for at least 3 months, rated their pain level over the last week as 4 out of 10 or higher, and had at least one day in the last month where pain made it hard to perform their usual daily activities.

STEPS and RESET participants were recruited primarily through health fairs, information sessions at senior centers and senior housing facilities, social media, and letters through a partnering healthcare system (for STEPS only). Participants in both studies provided verbal informed consent over the phone, with the option to receive the consent document by mail or email to review ahead of time, or by mail after verbal consent. Research staff read the consent form aloud, paused after each section for questions, and audio-recorded this process with participants' permission. Participants earned up to $65 for completing all surveys, and control group participants were offered a version of the STEPS or RESET intervention after their final survey.

Demographic and pain data were collected during screening or baseline assessments. Social needs screener questions were administered in-person or via telephone during participants' first meeting with a community health worker. This meeting took place after randomization and before starting the intervention for the STEPS and RESET intervention group members. RESET control group members were administered the screener in a post-randomization “wellness check” phone call. STEPS control group members did not receive the social needs screener unless they opted to participate in a one-time pain management workshop following final data collection; data from this group are not used in the present analysis. Participants were provided with resource referrals to help address unmet needs. Follow-up data were collected for both studies (at 2 and 12 months for STEPS and 2 and 8 months for RESET) but are not used in the present analysis.

### 2.2 Predictors

Social needs predictors were drawn from standard screeners related to the social determinants of health (30), and were restricted to domains for which the community health worker could readily provide resources. Items were measured as no = 0 and yes = 1, and were as follows: (1) “Do you need support with other health or medical expenses like vision, dental, or medical equipment?”; (2) “Are you experiencing any challenges related to getting food?”; (3) “Do you need help with housing or housing concerns?”; (4) “Do you have a hard time paying your utility bills?”; (5) “Do you have access to the internet/Wi-Fi where you live?” (reverse-coded); (6) “Do you need help finding or paying for care for yourself or a loved one?”; (7) “Do you have trouble with transportation?”; and (8) “Do you have the social support that you need?” (reverse-coded). Each item was retained as a binary indicator and a cumulative unmet social needs score was also created by summing the eight items (range 0–8). Given the skewed distribution of cumulative unmet social needs, with fewer participants reporting three or more needs, and consistent with other studies (31, 32), cumulative unmet needs was collapsed into four categories: 0, 1, 2, and ≥3 unmet needs.

### 2.3 Outcomes

Three pain outcomes were examined. *Pain intensity* was assessed as average pain in the past 7 days on an 11-point Numerical Rating Scale (0 = no pain, 10 = worst imaginable pain) (33). The standardized Patient-Reported Outcomes Measurement Information System (PROMIS) *Pain Interference* 4-item short form assessed the extent to which pain interfered with daily activities in the past 7 days, measured on a 5-point Likert scale (1 = not at all, 5 = very much) (34). Items were summed and converted into T-scores using conversion tables (35); T-scores have a population mean of 50 and standard deviation of 10. The 2-item version of the *Pain Self-Efficacy Questionnaire* (PSEQ) was scored by summing responses to the items: “I can do some form of work despite pain” and “I can live a normal lifestyle despite the pain” (7-point Likert scale: 0 = not at all confident, 6 = completely confident) (36). The PSEQ-2 was collected in the STEPS study only.

### 2.4 Covariates

Covariates included: *age* (years), *gender*, *race* (Black or African American, White, Other), *education* [high school graduate/General Educational Development (GED) or less, some postsecondary education (including vocational/technical, some college, or associate's degree), and bachelor's degree or higher] and number of self-reported *chronic conditions* (whether a doctor or other healthcare professional had ever diagnosed arthritis, low back pain, high blood pressure, diabetes, heart attack, angina, high cholesterol, depression, cancer, stroke, asthma, chronic obstructive pulmonary disease, migraine, heartburn, or osteoporosis). One participant self-identifying as transgender male (Other) was coded as male in regression analyses.

### 2.5 Analysis

Sample characteristics were summarized using means and standard deviations for continuous variables and counts and percentages for categorical variables. Bivariate tests assessed the relationships between cumulative unmet social needs or individual needs and pain outcomes (pain intensity, pain interference, pain self-efficacy). Analysis of variance (ANOVA) was used to compare mean scores of pain outcomes across the four levels of cumulative unmet social needs (0, 1, 2, and ≥3), and Tukey's honestly significant difference (HSD) was applied to identify significant pairwise differences. Individual unmet needs of interest (food, housing, utilities, transportation, and social support) were each treated as a binary indicator (no, yes), and mean pain outcome scores were compared between groups using Welch's *t*-tests.

Separate linear regression models examined the associations of cumulative unmet social needs and individual social needs (food, housing, utilities, transportation, and insufficient social support) with each pain outcome. All regression models adjusted for age, gender, race, education, and number of chronic conditions. Analyses included participants with available data for each outcome (pain intensity: *n* = 436; pain interference: *n* = 437; pain self-efficacy: *n* = 191). Missing values for categorical covariates were retained in regression models by including a Missing category for race (*n* = 13), education (*n* = 7), and gender (*n* = 2), such that adjusted and unadjusted models used identical samples. Multicollinearity was assessed using generalized variance inflation factor and all adjusted values were under 1.2, suggesting no problematic multicollinearity. Analyses were conducted in R version 4.5.3 with *p* < .05 considered statistically significant.

## 3 Results

Among 438 participants, the mean age was 67.5 years (SD = 8.1). Most (87%, *n* = 380) were female, 77% identified as Black or African American (*n* = 338), and over half (54%, *n* = 237) had one or more unmet social needs (Table 1). The unmet needs most frequently reported were housing (20%, *n* = 89), transportation difficulties and health or medical expense needs (both 18%, *n* = 81), Difficulty paying utility bills (17%, *n* = 74), and food access challenges (16%, *n* = 70). Fewer participants reported insufficient social support (11%, *n* = 46), needing assistance with childcare or eldercare (6%, *n* = 25), or internet access needs (5%, *n* = 24).

**Table 1:** Sample characteristics (*N* = 438).

Characteristics		Total sample (*N* = 438), *n* (%)
Age (years), Mean ± SD		67.5 ± 8.11
Gender	Female	380 (86.8)
	Male	55 (12.6)
	Other	1 (0.2)
	Missing	2 (0.5)
Marital status	Not married/partnered	347 (79.2)
	Married/partnered	85 (19.4)
	Missing	6 (1.4)
Race	Black or African American	338 (77.2)
	White	47 (10.7)
	Indigenous	2 (0.5)
	Multiple Races	37 (8.4)
	Other	1 (0.2)
	Missing	13 (3.0)
Ethnicity	Not Hispanic or Latino/a	424 (96.8)
	Hispanic or Latino/a	8 (1.8)
	Missing	6 (1.4)
Education	High school graduate/GED or less	64 (14.6)
	Some postsecondary education (Vocational/technical, some college, associate's degree)	202 (46.1)
	Bachelor's degree or higher (Bachelor's/graduate degree/PhD)	165 (37.7)
	Missing	7 (1.6)
Employment	Full time	50 (11.4)
	Part time	27 (6.2)
	Disability	16 (3.7)
	Retired	276 (63.0)
	Unemployed	62 (14.2)
	Other	2 (0.5)
	Missing	5 (1.1)
Cumulative number of unmet social needs	0 unmet need	201 (45.9)
	1 unmet need	108 (24.7)
	2 unmet needs	54 (12.3)
	≥3 unmet needs^a^	75 (17.1)
Individual unmet social needs	Food access challenges (Yes)	70 (16.0)
	Housing needs (Yes)	89 (20.3)
	Difficulty paying utility bills (Yes)	74 (16.9)
	Health or medical expense needs (Yes)	81 (18.5)
	Transportation difficulties (Yes)	81 (18.5)
	Internet access needs (Yes)	24 (5.5)
	Insufficient social support (Yes)	46 (10.5)
	Childcare or eldercare needs (Yes)	25 (5.7)
Number of chronic conditions (0–15), Mean ± SD		5.33 ± 2.27
Pain intensity (range 0 to 10, *n* = 436), Mean ± SD		5.78 ± 2.22
Pain interference (T-scores; population mean of 50, *n* = 437), Mean ± SD^b^		59.9 ± 8.09
Total pain self-efficacy (range 0–12, *n* = 191), Mean ± SD^c^		8.24 ± 3.36
I can do some form of work despite pain, Mean ± SD^c^		4.25 ± 1.78
I can live a normal lifestyle despite the pain, Mean ± SD^c^		3.99 ± 1.90

a Includes participants with three (*n* = 41), four (*n* = 22), five (*n* = 9) or six needs (*n* = 3).

b Higher scores indicate worse pain interference in daily life. Items were summed and converted into T-scores.

c Higher scores indicate increased self-efficacy in functioning in the presence of pain; data only available for STEPS dataset.

### 3.1 Bivariate associations between unmet social needs and pain outcomes

Bivariate tests examined associations between pain outcomes and cumulative and individual unmet social needs (Table 2). Cumulative unmet social needs were significantly associated with pain intensity (*p* = .002), pain interference (*p* < .001), and pain self-efficacy (*p* = .013). Tukey's HSD *post-hoc* comparisons indicated that participants with three or more unmet social needs reported significantly higher mean pain intensity than those without unmet needs (M = 6.61 vs. 5.49, *p* = .001) or one unmet need (M = 6.61 vs. 5.71, *p* = .033); the comparison with those reporting two unmet needs did not reach statistical significance for any outcome. A similar pattern emerged for pain interference *post-hoc* tests: participants reporting three or more unmet needs had worse interference than those with none (*M* = 63.26 vs. 58.65, *p* < .001) or one unmet need (*M* = 63.26 vs. 59.66, *p* = .015). Lastly, participants reporting three or more unmet social needs had lower pain self-efficacy than those with none (*M* = 6.15 vs. 8.69, *p* = .009).

**Table 2:** Bivariate associations between social need domains and pain outcomes.

Unmet social need		Pain intensity (*n* = 436)		Pain interference (*n* = 437)		Pain self-efficacy (*n* = 191)^a^	
		Mean ± SD	*P*-value	Mean ± SD	*P*-value	Mean ± SD	*P*-value
Cumulative number of unmet social need(s)^b^	0	5.49 ± 2.12	.002	58.65 ± 7.84	<.001	8.69 ± 3.31	.013
	1	5.71 ± 2.26		59.66 ± 8.34		7.78 ± 3.23	
	2	5.87 ± 2.36		60.70 ± 7.38		8.53 ± 2.53	
	≥3	6.61 ± 2.16		63.26 ± 8.06		6.15 ± 3.75	
Food access challenges	No	5.57 ± 2.21	<.001	59.37 ± 8.11	<.001	8.39 ± 3.29	.081
	Yes	6.93 ± 1.87		62.92 ± 7.35		6.65 ± 3.76	
Housing needs	No	5.67 ± 2.25	.033	59.55 ± 8.25	.034	8.32 ± 3.31	.417
	Yes	6.21 ± 2.06		61.46 ± 7.28		7.69 ± 3.67	
Difficulty paying utility bills	No	5.79 ± 2.12	.920	59.78 ± 7.79	.434	8.33 ± 3.32	.399
	Yes	5.76 ± 2.67		60.70 ± 9.43		7.75 ± 3.57	
Transportation difficulties	No	5.65 ± 2.23	.006	59.40 ± 8.13	.002	8.46 ± 3.34	.005
	Yes	6.38 ± 2.09		62.32 ± 7.52		6.21 ± 2.94	
Insufficient social support	No	5.68 ± 2.23	.002	59.52 ± 8.11	.001	8.37 ± 3.30	.042
	Yes	6.67 ± 1.89		63.55 ± 7.03		5.44 ± 3.61	

a Dataset contains participants from the STEPS study only.

b ANOVA; Tukey's *post-hoc* test results are not reported in the table. All other results describe t-tests.

Regarding individual social need domains, transportation difficulties and insufficient social support were associated with higher pain intensity and interference, and lower self-efficacy (all *p* < .05). Housing needs and food access challenges were significantly associated with higher pain intensity and interference but were not associated with pain self-efficacy. Difficulty paying utility bills were not significantly associated with any pain outcome (all *p* > .05).

### 3.2 Multivariable regressions examining unmet social needs and pain outcomes

In regression models adjusted for age, gender, race, education, and number of chronic conditions (Table 3), participants reporting three or more unmet social needs had significantly higher pain intensity (*B* = 0.75, *p* = .010, 95% CI: 0.18, 1.32), pain interference (*B* = 3.24, *p* = .002, 95% CI: 1.24, 5.23) and lower pain self-efficacy (*B* = −2.04, *p* = .006, 95% CI: −3.50, −0.59) compared to those without unmet social needs.

**Table 3:** Multivariable regression results for unmet social needs and pain intensity, interference, and self-efficacy. Models adjusted for age, gender, race, education, and number of chronic conditions.

Unmet social need		Pain intensity (*n* = 436)			Pain interference (*n* = 437)			Pain self-efficacy^a^ (*n* = 191)		
		B	95% CI	*P*-value	B	95% CI	*P*-value	B	95% CI	*P*-value
Cumulative number of unmet social need(s)^b^	1	0.13	−0.36, 0.62	.613	0.71	−1.00, 2.41	.415	−1.09	−2.23, 0.05	.061
	2	0.07	−0.56, 0.71	.818	1.13	−1.07, 3.33	.312	−0.53	−2.16, 1.11	.525
	≥3	0.75	0.18, 1.32	.010	3.24	1.24, 5.23	.002	−2.04	−3.50, −0.59	.006
									
Food access challenges^b^		1.08	0.54, 1.61	<.001	2.53	0.65, 4.42	.009	−1.54	−3.07, −0.01	.048
									
Housing needs^b^		0.39	−0.10, 0.89	.119	1.60	−0.14, 3.34	.072	−0.71	−2.00, 0.57	.274
									
Difficulty paying utility bills^b^		−0.22	−0.75, 0.32	.428	0.45	−1.41, 2.32	.634	−0.55	−1.72, 0.63	.361
									
Transportation difficulties^b^		0.47	−0.05, 0.98	.076	2.06	0.25, 3.86	.026	−1.82	−3.27, −0.38	.014
									
Insufficient social support^c^		0.74	0.10, 1.38	.024	2.44	0.17, 4.71	.035	−1.90	−3.97, 0.16	.071

a Only available in STEPS data.

b Reference: None reported.

c Reference: Sufficient social support.

In adjusted models examining individual needs, participants with food access challenges or insufficient social support reported higher pain intensity than those without these challenges (food access: *B* = 1.08, *p* < .001, 95% CI: 0.54, 1.61; social support: *B* = 0.74, *p* = .024, 95% CI: 0.10, 1.38). Those with food access challenges, transportation difficulties, or insufficient social support reported greater pain interference than those without (food access: *B* = 2.53, *p* = .009, 95% CI: 0.65, 4.42; transportation: *B* = 2.06, *p* = .026, 95% CI: 0.25, 3.86; social support: *B* = 2.44, *p* = .035, 95% CI: 0.17, 4.71). Participants reporting food access challenges or transportation difficulties also had lower pain self-efficacy compared to those without these challenges (food access: *B* = −1.54, *p* = .048, 95% CI: −3.07, −0.01; transportation difficulties: *B* = −1.82, *p* = .014, 95% CI: −3.27, −0.38). There were no significant relationships between housing or utility bill payment difficulties and any pain outcome, transportation difficulties and pain intensity, or insufficient social support and pain self-efficacy (*p* > .05). Unadjusted model results are included in Supplementary Material 1.

## 4 Discussion

This study examined associations between unmet social needs and pain outcomes among midlife and older adults with chronic pain in an underserved urban community. More than half the sample reported at least one unmet need; most commonly housing, health or medical expenses, and/or transportation. In adjusted regression models, only the highest category of cumulative unmet social needs (three or more versus none) was significantly associated with worse pain outcomes. This pattern may reflect co-occurring challenges that deplete the time and resources needed for pain self-management (37). Studies have reported that lower income is associated with worse pain self-efficacy scores (38) and there is an association between a greater number of unmet social needs and lower health confidence (39). More unmet social needs have been associated with higher perceived stress (40), which may in turn exacerbate pain. Together, these co-occurring challenges reflect a high level of disadvantage that may act as a fundamental cause of poor health (41, 42).

This stress pathway is exemplified in the weathering hypothesis, which proposes that chronic psychosocial stressors, including structural racism and economic hardship, contribute to physiological stress dysregulation and accelerated aging (28, 43); this dysregulation has been linked to increased risk of chronic pain (26, 27). This framework may be relevant to our sample: participants lived in or near Detroit, a city that has experienced structural inequities, including residential segregation, redlining, and disinvestment (28, 44). Most participants in this sample identified as Black or African American, a population disproportionately impacted by such policies and practices. However, experiences of stress are heterogeneous, shaped by intersecting social identities, life course exposures, and access to protective social resources and community supports (28).

Among individual social need domains, transportation challenges were associated with pain interference and self-efficacy. Pain self-efficacy is an important, modifiable target for behavioral interventions to improve pain management (45). This novel finding suggests that unaddressed transportation difficulties may undermine pain interventions. Primary care patients experiencing depression and pain have reported that limited transportation and financial resources, as well as inadequate social support, are barriers to pain self-management (37). These barriers are particularly salient for older adults, who may have greater healthcare needs, restricted mobility, or may no longer drive (46, 47). Transportation also likely intersects with other domains, including food access (48) and social support, which could exacerbate pain disparities.

Food access challenges showed the largest magnitude of association with pain intensity across individual social needs and were also associated with pain interference and self-efficacy, although the self-efficacy association should be interpreted with caution (*p* = .048). These results corroborate nationally representative evidence that food insecurity is associated with increased odds of pain among older adults (24). Mechanistically, food access challenges may worsen bodily function through obesity, insufficient nutrition, and inflammation (24, 49). The stress of insufficient food access or any unmet social need may also exacerbate pain, consistent with the stress pathways described previously. Insufficient social support was associated with pain intensity and interference, consistent with research findings that social support is directly linked to decreased pain intensity (50). Social support is a well-established protective factor against pain, operating through improved coping (51) and mental health, as well as reduced physiological stress responses to pain (50, 52).

In contrast, challenges related to housing and utility bills were not associated with any pain outcome in adjusted models. This finding aligns with a Midwestern study on emergency department patients that reported no significant associations between housing instability and pain intensity, interference, and self-efficacy (53). Alternatively, our screening item for housing needs may have lacked specificity regarding the type or severity of housing challenges. Future research should use more granular measures of housing circumstances to better assess these relationships.

This study addresses a gap in the literature by examining multiple social needs in relation to pain outcomes among midlife and older adults. Prior research has largely examined single social factors and pain in isolation, limiting our understanding of how multiple needs relate to pain. Unmet social needs arise from intersecting inequities, including structural racism, that can disproportionately shape the conditions and opportunities for Black, Indigenous, and other people of color (17) as well as economic stratification in society. Therefore, preventing and addressing chronic pain disparities requires multi-level interventions, including structural reform that honors community strengths, while addressing the root causes of inequitable resource distribution and discrimination in pain treatment. Understanding differences in the experiences and treatment of these adults can inform provider training on whole-person care, non-discriminatory clinical practice, and the development of culturally affirmed pain management interventions (54, 55).

Further, our findings have important implications for clinical practice. In this sample of adults living in a city shaped by structural inequities, more than half of participants with chronic pain experienced at least one unmet social need. While pain care seldom focuses on the social aspects of pain (17), these results underscore the need for greater integration of social needs screening and interventions into pain care. This may occur by embedding community health workers and/or social workers into interdisciplinary pain care models for improved pain management (56–58).

Beyond clinical care, these findings underscore the importance of programs and policies that prevent and address unmet social needs among midlife and older adults. While such programs, including the Supplemental Nutrition Assistance Program and food prescriptions, have been evaluated for health and/or economic outcomes (e.g., 59–61) their impact on pain is not well-established. Future evaluation of such programs should consider including multiple dimensions of pain experience as outcomes.

Study findings should be interpreted alongside limitations. This cross-sectional design cannot establish the directionality between social needs and pain outcomes; nor were we able to determine whether unmet needs directly affect pain or are indicators of another dimension of socioeconomic position that affects pain. For example, chronic pain and disability may contribute to changes in employment and financial burden (62). Additionally, the binary response format for social need questions may obscure heterogeneity in the severity of social needs. For example, participants indicating they need assistance with housing may be describing houselessness or home repairs. This variation may influence the association between social needs and pain outcomes. Similarly, participants reporting three or more unmet needs were collapsed into a single cumulative unmet need category, which obscures variation in needs within that group. The findings may also not be generalizable to other settings or age groups. Future studies should examine these associations using nationally representative samples with longitudinal designs.

Overall, the findings indicate that cumulative unmet social needs, food access challenges, transportation difficulties, and insufficient social support were associated with multiple pain outcomes. Future research should assess whether interventions targeting unmet social needs improve pain outcomes longitudinally, with particular attention to midlife and older adults with multiple co-occurring unmet social needs.

## Data Availability

De-identified study data will be made publicly available via the Inter-university Consortium for Political and Social Research data repository upon study completion.

## References

[1] NCHS: Data Query System Application. Regularly experienced chronic pain. CDC Natl Cent Health Stat. (2025) Available online at: https://www.cdc.gov/nchs/dqs/index.html (Accessed April 15, 2026)

[2] Treede R-D Rief W Barke A Aziz Q Bennett MI Benoliel R. A classification of chronic pain for ICD-11. Pain. (2015) 156:1003–7. 10.1097/j.pain.0000000000000160 25844555 PMC4450869

[3] Ritchie CS Patel K Boscardin J Miaskowski C Vranceanu A-M Whitlock E. Impact of persistent pain on function, cognition, and well-being of older adults. J Am Geriatr Soc. (2023) 71:26–35. 10.1111/jgs.18125 36475388 PMC9871006

[4] Mookerjee N Schmalbach N Antinori G Thampi S Windle-Puente D Gilligan A. Association of risk factors and comorbidities with chronic pain in the elderly population. J Prim Care Community Health. (2024) 15:21501319241233463. 10.1177/21501319241233463 38366930 PMC10874592

[5] Bell T Franz CE Kremen WS. Persistence of pain and cognitive impairment in older adults. J Am Geriatr Soc. (2022) 70:449–58. 10.1111/jgs.17542 34741304 PMC8821128

[6] Yang S Wang H Tao Y Tian J Wen Z Cao J. Association of chronic pain with frailty and pre-frailty in older adults: a systematic review and meta-analysis. Arch Gerontol Geriatr. (2025) 131:105784. 10.1016/j.archger.2025.105784 39954602

[7] Camacho D Burnette D Aranda MP Moxley JH Lukens EP Reid MC. Loneliness and pain among community-dwelling middle-aged and older black, Latino, and white adults in the United States. Front Public Health. (2024) 12:54. 10.3389/fpubh.2024.1429739 PMC11457733 39377004

[8] Ryan E Grol-Prokopczyk H Dennison CR Zajacova A Zimmer Z. Is the relationship between chronic pain and mortality causal? A propensity score analysis. PAIN. (2025) 166:183. 10.1097/j.pain.0000000000003336 38981067 PMC11647826

[9] Macfarlane GJ Barnish MS Jones GT. Persons with chronic widespread pain experience excess mortality: longitudinal results from UK biobank and meta-analysis. Ann Rheum Dis. (2017) 76:1815–22. 10.1136/annrheumdis-2017-211476 28733474

[10] Gignac MAM Davis AM Hawker G Wright JG Mahomed N Fortin PR. “What do you expect? You’re just getting older”: a comparison of perceived osteoarthritis-related and aging-related health experiences in middle- and older-age adults. Arthritis Care Res. (2006) 55:905–12. 10.1002/art.22338 17139636

[11] Thielke S Sale J Reid MC. Aging: are these 4 pain myths complicating care? J Fam Pract. (2012) 61:666–70. 23256096 PMC4356472

[12] Spector AL Quinn KG Cruz-Almeida Y Fillingim RB. Chronic pain among middle-aged and older adults in the United States: the role of everyday discrimination and racial/ethnic identity. J Pain. (2024) 25:104439. 10.1016/j.jpain.2023.11.022 38065467 PMC11058034

[13] Rambachan A Neilands TB Karliner L Covinsky K Fang M Nguyen T. Pain management inequities by demographic and geriatric-related variables in older adult inpatients. J Am Geriatr Soc. (2024) 72:3000–10. 10.1111/jgs.19076 38997213 PMC11844259

[14] Spector AL. Pain management for older adults in disadvantaged communities. Clin Geriatr Med. (2026) 42:151–60. 10.1016/j.cger.2025.08.009 41271279

[15] Morales ME Yong RJ. Racial and ethnic disparities in the treatment of chronic pain. Pain Med. (2021) 22:75–90. 10.1093/pm/pnaa427 33367911

[16] Grol-Prokopczyk H Huang R Yu C Chen Y-A Kaur S Limani M. Over 50 years of research on social disparities in pain and pain treatment: a scoping review of reviews. Pain. (2025) 166:2458–72. 10.1097/j.pain.0000000000003676 40553634 PMC12353892

[17] Kapos FP Craig KD Anderson SR Bernardes SF Hirsh AT Karos K. Social determinants and consequences of pain: toward multilevel, intersectional, and life course perspectives. J Pain. (2024) 25:104608. 10.1016/j.jpain.2024.104608 38897311 PMC11402600

[18] Li C Liu C Ye C Lian Z Lu P. Education, gender, and frequent pain among middle-aged and older adults in the United States, England, China, and India. PAIN. (2025) 166:388–97. 10.1097/j.pain.0000000000003349 39190366

[19] Ulirsch JC Weaver MA Bortsov AV Soward AC Swor RA Peak DA. No man is an island: living in a disadvantaged neighborhood influences chronic pain development after motor vehicle collision. PAIN. (2014) 155:2116. 10.1016/j.pain.2014.07.025 25107859 PMC4197076

[20] Khalatbari-Soltani S Blyth FM. Socioeconomic position and pain: a topical review. PAIN. (2022) 163:1855. 10.1097/j.pain.0000000000002634 35297800

[21] Fuentes M Hart-Johnson T Green CR. The association among neighborhood socioeconomic status, race and chronic pain in black and white older adults. J Natl Med Assoc. (2007) 99:1160–9. 17987920 PMC2574394

[22] Lacey RJ Belcher J Croft PR. Does life course socio-economic position influence chronic disabling pain in older adults? A general population study. Eur J Public Health. (2013) 23:534–40. 10.1093/eurpub/cks056 22874735 PMC3719471

[23] Kreuter MW, Thompson T, McQueen A, Garg R. Addressing social needs in health care settings: evidence, challenges, and opportunities for public health. Annu Rev Public Health. (2021) 42:329–44. 10.1146/annurev-publhealth-090419-102204 33326298 PMC8240195

[24] Tamargo JA Strath LJ Karanth SD Spector AL Sibille KT Anton S. Food insecurity is associated with chronic pain and high-impact chronic pain in the USA. Public Health Nutr. (2023) 27:e7. 10.1017/S1368980023002732 38087858 PMC10830368

[25] Karayannis NV Baumann I Sturgeon JA Melloh M Mackey SC. The impact of social isolation on pain interference: a longitudinal study. Ann Behav Med Publ Soc Behav Med. (2019) 53:65–74. 10.1093/abm/kay017 PMC6301311 29668841

[26] Liang Y Booker C. Allostatic load and chronic pain: a prospective finding from the national survey of midlife development in the United States, 2004–2014. BMC Public Health. (2024) 24:416. 10.1186/s12889-024-17888-1 38336697 PMC10854121

[27] Hobson JM Moody MD Sorge RE Goodin BR. The neurobiology of social stress resulting from racism: implications for pain disparities among racialized minorities. Neurobiol Pain. (2022) 12:100101. 10.1016/j.ynpai.2022.100101 36092741 PMC9449662

[28] Geronimus A Pearson J Linnenbringer E Eisenberg A Stokes C Hughes L. Weathering in Detroit: place, race, ethnicity, and poverty as conceptually fluctuating social constructs shaping variation in allostatic load. Milbank Q. (2020) 98:1171–218. 10.1111/1468-0009.12484 33135829 PMC7772642

[29] Janevic MR Lindsay R Brines E Wisdom K Robinson-Lane SG Brewer R. A community health worker-delivered intervention (STEPS) to support chronic pain self-management among older adults in an underserved urban community: protocol for a randomized trial. Trials. (2025) 26:186. 10.1186/s13063-025-08892-w 40457457 PMC12128255

[30] Moen M Storr C German D Friedmann E Johantgen M. A review of tools to screen for social determinants of health in the United States: a practice brief. Popul Health Manag. (2020) 23:422–9. 10.1089/pop.2019.0158 31910355 PMC7864106

[31] Heller CG Rehm CD Parsons AH Chambers EC Hollingsworth NH Fiori KP. The association between social needs and chronic conditions in a large, urban primary care population. Prev Med. (2021) 153:106752. 10.1016/j.ypmed.2021.106752 34348133 PMC8595547

[32] Zwaschka T Sebesta E Gleicher S Kaufman M Dmochowski R Reynolds W. The cumulative effect of unmet social needs on noncancerous genitourinary conditions and severity of lower urinary tract symptoms. Neurourol Urodyn. (2022) 41:1862–71. 10.1002/nau.25038 36066087 PMC9633428

[33] Hays RD Bjorner JB Revicki DA Spritzer KL Cella D. Development of physical and mental health summary scores from the patient-reported outcomes measurement information system (PROMIS) global items. Qual Life Res Int J Qual Life Asp Treat Care Rehabil. (2009) 18:873–80. 10.1007/s11136-009-9496-9 PMC2724630 19543809

[34] Cella D Choi SW Condon DM Schalet B Hays RD Rothrock NE. PROMIS® adult health profiles: efficient short-form measures of seven health domains. Value Health J Int Soc Pharmacoeconomics Outcomes Res. (2019) 22:537–44. 10.1016/j.jval.2019.02.004 PMC7201383 31104731

[35] Health Measures. Pain interference scoring manual. HealthMeasures.net. (2023) Available online at: https://www.healthmeasures.net/images/PROMIS/manuals/Scoring_Manual_Only/PROMIS_Pain_Interference_Scoring_Manual_05Dec2023.pdf (Accessed March 7, 2026).

[36] Nicholas MK McGuire BE Asghari A. A 2-item short form of the pain self-efficacy questionnaire: development and psychometric evaluation of PSEQ-2. J Pain. (2015) 16:153–63. 10.1016/j.jpain.2014.11.002 25463701

[37] Bair MJ Matthias MS Nyland KA Huffman MA Stubbs DWL Kroenke K. Barriers and facilitators to chronic pain self-management: a qualitative study of primary care patients with comorbid musculoskeletal pain and depression. Pain Med. (2009) 10:1280–90. 10.1111/j.1526-4637.2009.00707.x 19818038 PMC2884223

[38] Whitley MD Herman PM Aliyev GR Sherbourne CD Ryan GW Coulter ID. Income as a predictor of self-efficacy for managing pain and for coping with symptoms among patients with chronic low back pain. J Manipulative Physiol Ther. (2021) 44:433–44. 10.1016/j.jmpt.2021.05.004 34470698 PMC8492484

[39] Bleacher H English A Leblanc W Dickinson LM. Associations between Patients’ unmet social needs and self-reported health confidence at one primary care clinic. J Prim Care Community Health. (2020) 11:2150132720921329. 10.1177/2150132720921329 32410492 PMC7232046

[40] Thompson T McQueen A Croston M Luke A Caito N Quinn K. Social needs and health-related outcomes among medicaid beneficiaries. Health Educ Behav. (2019) 46:436–44. 10.1177/1090198118822724 30654655

[41] Link BG Phelan J. Social conditions as fundamental causes of disease. J Health Soc Behav. (1995) Spec No:80–94. 10.2307/2626958 7560851

[42] Phelan JC Link BG. Is racism a fundamental cause of inequalities in health? Annu Rev Sociol. (2015) 41:311–30. 10.1146/annurev-soc-073014-112305

[43] Guidi J Lucente M Sonino N Fava GA. Allostatic load and its impact on health: a systematic review. Psychother Psychosom. (2020) 90:11–27. 10.1159/000510696 32799204

[44] Mehdipanah R McVay KR Schulz AJ. Historic redlining practices and contemporary determinants of health in the detroit metropolitan area. Am J Public Health. (2023) 113:S49–57. 10.2105/AJPH.2022.307162 36696614 PMC9877378

[45] Schumann ME Coombes BJ Gascho KE Geske JR McDermott MC Morrison EJ. Pain catastrophizing and pain self-efficacy mediate interdisciplinary pain rehabilitation program outcomes at posttreatment and follow-up. Pain Med. (2022) 23:697–706. 10.1093/pm/pnab271 34519826

[46] Ruggiano N Shtompel N Whiteman K Sias K. Influences of transportation on health decision-making and self-management behaviors among older adults with chronic conditions. Behav Med. (2017) 43:61–70. 10.1080/08964289.2015.1065788 26207609

[47] Syed ST Gerber BS Sharp LK. Traveling towards disease: transportation barriers to health care access. J Community Health. (2013) 38:976–93. 10.1007/s10900-013-9681-1 23543372 PMC4265215

[48] Keyes LM Kim J Balachandran S Kuttler S Andrew S. Missing meals and missed rides: transportation barriers to food access for vulnerable populations. Urban Sci. (2025) 9:198. 10.3390/urbansci9060198

[49] Elma Ö Brain K Dong H-J. The importance of nutrition as a lifestyle factor in chronic pain management: a narrative review. J Clin Med. (2022) 11:5950. 10.3390/jcm11195950 36233817 PMC9571356

[50] Zhang X Zhang R Lin X Zeng L Zhang J. The impact of social support on pain intensity in older adults with chronic pain: the serial mediating roles of positive and negative affect and pain catastrophizing. BMC Geriatr. (2026) 26:289. 10.1186/s12877-026-07081-x 41629838 PMC12952191

[51] Jensen MP Moore MR Bockow TB Ehde DM Engel JM. Psychosocial factors and adjustment to chronic pain in persons with physical disabilities: a systematic review. Arch Phys Med Rehabil. (2011) 92:146–60. 10.1016/j.apmr.2010.09.021 21187217 PMC3028590

[52] Franqueiro AR Yoon J Crago MA Curiel M Wilson JM. The interconnection between social support and emotional distress among individuals with chronic pain: a narrative review. Psychol Res Behav Manag. (2023) 16:4389–99. 10.2147/PRBM.S410606 37915959 PMC10617401

[53] ShermanJP AlbrightN EmersonB CobranchiC BrownJL MorganE. No place to rest: housing instability and pain experiences in the emergency department. J Emerg Nurs. (2026) 52:513–26. 10.1016/j.jen.2025.12.00341553306

[54] Baker TA Booker SQ Janevic MR. A progressive agenda toward equity in pain care. Health Psychol Behav Med. (2023) 11:2266221. 10.1080/21642850.2023.2266221 37818413 PMC10561565

[55] Baker TA Clay OJ Johnson-Lawrence V Minahan JA Mingo CA Thorpe RJ. Association of multiple chronic conditions and pain among older black and white adults with diabetes mellitus. BMC Geriatr. (2017) 17:255. 10.1186/s12877-017-0652-8 29084525 PMC5663150

[56] Colón Y Otis-Green S. Roles of social workers in interdisciplinary pain management. J Pain Palliat Care Pharmacother. (2008) 22:303–5. 10.1080/15360280802537308 21923315

[57] Hooten WM Backonja M Williams KA Sturgeon JA Gross JB Borodianski S. Integrated pain care models and the importance of aligning stakeholder values. Pain Rep. (2024) 9:e1160. 10.1097/PR9.0000000000001160 38646660 PMC11029933

[58] Brecht DM Stephens J Gatchel RJ. Interdisciplinary pain management programs in the treatment of pain conditions. In: Pain Management for Clinicians: A Guide to Assessment and Treatment. Noe CE, editors. Cham: Springer International Publishing (2020). p. 461–89. 10.1007/978-3-030-39982-5_18

[59] Ratcliffe C McKernan S-M Zhang S. How much does the supplemental nutrition assistance program reduce food insecurity? Am J Agric Econ. (2011) 93:1082–98. 10.1093/ajae/aar026 25197100 PMC4154696

[60] Hoynes H Schanzenbach DW Almond D. Long-Run impacts of childhood access to the safety net. Am Econ Rev. (2016) 106:903–34. 10.1257/aer.20130375

[61] Hager K Du M Li Z Mozaffarian D Chui K Shi P. Impact of produce prescriptions on diet, food security, and cardiometabolic health outcomes: a multisite evaluation of 9 produce prescription programs in the United States. Circ Cardiovasc Qual Outcomes. (2023) 16:e009520. 10.1161/CIRCOUTCOMES.122.009520 37641928 PMC10529680

[62] Halpern MT De Moor JS Yabroff KR. Impact of pain on employment and financial outcomes among cancer survivors. J Clin Oncol. (2022) 40:24–31. 10.1200/JCO.20.03746 34292791 PMC9851709

