# Supplementary material for: Unmet health-related social needs are associated with pain outcomes among midlife and older adults
Source: Front Pain Res (Lausanne). 2026 Sep 1;7:1885908. doi: 10.3389/fpain.2026.1885908 (PMC13574968; doi:10.3389/fpain.2026.1885908)
Supplement: Supplementary file 1 [file Table1.pdf]

Supplementary Material 1. Unadjusted regression results for cumulative and individual unmet social needs and pain intensity, interference, and self-efficacy

		**Pain intensity (n=436)**			**Pain interference (n=437)**			**Pain self-efficacy (n=191)[a]**		
		B	95% CI	*P*-value	B	95% CI	*P*-value	B	95% CI	*P*-value
**Cumulative number of unmet social need(s)[b]**	1	0.23	−0.29, 0.74	.389	1.00	−0.86, 2.87	.291	−0.91	−2.13, 0.32	.146
	2	0.38	−0.28, 1.04	.255	2.05	−0.35, 4.44	.094	−0.16	−1.93, 1.62	.863
	≥3	1.13	0.54, 1.71	<.001	4.60	2.48, 6.73	<.001	−2.54	−4.11, −0.97	.002
**Food access challenges[b]**		1.36	0.81, 1.92	<.001	3.55	1.51, 5.60	<.001	−1.74	−3.41, −0.07	.041
**Housing needs[b]**		0.54	0.02, 1.06	.041	1.91	0.02, 3.80	.047	−0.63	−2.03, 0.77	.377
**Difficulty paying utility bills[b]**		−0.03	−0.59, 0.52	.906	0.92	−1.11, 2.95	.374	−0.58	−1.87, 0.70	.372
**Transportation difficulties[b]**		0.73	0.20, 1.27	.007	2.92	0.97, 4.87	.003	−2.25	−3.82, −0.67	.005
**Insufficient social support[c]**		0.99	0.32, 1.67	.004	4.03	1.55, 6.51	.001	−2.93	−5.16, −0.70	.010

[a]Only available in STEPS data
[b]Reference: None reported
[c]Reference: Sufficient social support
